# Association of *phospholipase A2 receptor 1 *polymorphisms with idiopathic membranous nephropathy in Chinese patients in Taiwan

**DOI:** 10.1186/1423-0127-17-81

**Published:** 2010-10-11

**Authors:** Yu-Huei Liu, Cheng-Hsu Chen, Shih-Yin Chen, Ying-Ju Lin, Wen-Ling Liao, Chang-Hai Tsai, Lei Wan, Fuu-Jen Tsai

**Affiliations:** 1 Department of Medical Genetics and Medical Research, China Medical University Hospital, Taichung, Taiwan; 2 School of Chinese Medicine, China Medical University, Taichung, Taiwan; 3 Division of Nephrology, Department of Internal Medicine, Taichung Veterans General Hospital, Taichung, Taiwan; 4 Department of Pediatrics, China Medical University Hospital, Taichung, Taiwan; 5 Asia University, Taichung, Taiwan; 6 Department of Health and Nutrition Biotechnology, Asia University, Taichung, Taiwan; 7 School of Post-Baccalaureate Chinese Medicine, China Medical University, Taichung, Taiwan; 8 Department of Biotechnology, Asia University, Taichung, Taiwan; 9 Department of Biotechnology and Bioinformatics, Asia University, Taichung, Taiwan

## Abstract

**Background:**

Idiopathic membranous nephropathy (IMN) is one of the most common forms of autoimmune nephritic syndrome in adults. The purpose of this study is to evaluate whether polymorphisms of *PLA2R1 *affect the development of IMN.

**Methods:**

Taiwanese-Chinese individuals (129 patients with IMN and 106 healthy controls) were enrolled in this study. The selected single nucleotide polymorphisms (SNPs) in *PLA2R1 *were genotyped by real-time polymerase chain reaction using TaqMan fluorescent probes, and were further confirmed by polymerase chain reaction-restriction fragment length polymorphism. The roles of the SNPs in disease progression were analyzed.

**Results:**

Genotype distribution was significantly different between patients with IMN and controls for *PLA2R1 *SNP rs35771982 (*p *= 0.015). The frequency of *G *allele at rs35771982 was significantly higher in patients with IMN as compared with controls (*p *= 0.005). In addition, haplotypes of *PLA2R1 *may be used to predict the risk of IMN (*p *= 0.004). Haplotype H1 plays a role in an increased risk of IMN while haplotype H3 plays a protective role against this disease. None of these polymorphisms showed a significant and independent influence on the progression of IMN and the risk of end-stage renal failure and death (ESRF/death). High disease progression in patients having *C/T *genotype at rs6757188 and *C/G *genotype at rs35771982 were associated with a low rate of remission.

**Conclusions:**

Our results provide new evidence that genetic polymorphisms of *PLA2R1 *may be the underlying cause of IMN, and the polymorphisms revealed by this study warrant further investigation.

## Background

Podocytes are highly specialized cells that play a crucial role in the glomerular filtration barrier [1,2]. Alterations in surface molecules of podocytes lead to the accumulation of antipodocytic antibodies on podocytes within the kidney, which leads to autoimmune response and cell damage [3]. When damage occurs, the interaction between immune-related factors and damaged cells can lead to foot process retraction, proteinuria, destruction of the filtration barrier, nephritis, and subsequently induction of end-stage renal failure and death (ESRF/death) [4,5].

Accumulating evidence suggests that podocytes are the primary target of injury in renal glomerular diseases [6-8]. Membranous nephropathy (MN) is one of the most common forms of nephrotic syndrome in adults, accounting for 40% of cases of ESRF; it occurs after 10 years from the time of the initial diagnosis of the disease [9]. One of its subtypes, idiopathic MN (IMN), is an autoimmune disease that represents approximately 75% of MN cases. This disease is characterized by thickening of the basement membrane and subepithelial immune deposits without cellular proliferation or infiltration [9]. Therapies for IMN that include the use of immunosuppressive drugs and nonspecific antiproteinuric measures have led to disappointing results and prompted increased interest in the discovery of new therapeutic targets [10].

Phospholipase A_2 _receptor 1 *(*PLA2R1) belongs to the mannose receptor family and is a type I transmembrane glycoprotein (~180 kDa). It is composed of a large extracellular portion that consists of an N-terminal cysteine-rich region, a fibronectin-like type II domain, a tandem repeat of 8 C-type lectin domains (CTLDs), a transmembrane domain, and a short intracellular C-terminal region [11,12]. PLA2R1 is known to regulate a variety of biological responses that are elicited by secretory phospholipase A2 (sPLA2) [11]. A recent study identified antibodies specific to PLA2R1 in 70% (26 of 37) of patients with IMN [13]. Although PLA2R1 has been proposed as the target autoantigen in IMN, the relationship between the polymorphisms of *PLA2R1 *and the development of this disease has not yet been examined. In this study, we analyzed the gene polymorphisms of *PLA2R1 *by using TaqMan allele discrimination, and examined their possible role in IMN.

## Methods

### Patients and controls

A disease group composed of 129 patients with biopsy-diagnosed IMN and a gender-matched control group composed of 106 healthy individuals, identified through health examination at Taichung Veterans General Hospital in Taiwan, were enrolled. All participants in this study provided informed consent as approved by the ethics committee of Taichung Veterans General Hospital. Patients and controls are 85% Minnan descendants; 5% Hakka descendants; and 10% mixed population of Minnan, Hakka, and Canton descendants.

#### Patients

Patients with any evidence of secondary causes such as malignancy, chronic infection with hepatitis B and C viruses, lupus nephritis, or other specific diseases, were excluded. The average age of patients was 59.8 (±17.4) years for men and 53.7 (± 15.6) years for women; average body mass index (BMI) was 24.8 (± 3.6); 62 (48.1%) patients were hypertensive, with blood pressure of >140/90 mm Hg; 21 (16.3%) patients have ESRF requiring renal replacement therapy; and 12 (9.3%) patients died from IMN. The clinical data were collected from patients at regular intervals as follows: the plasma creatine was measured by UniCel DxC 800 PRO Synchron clinical chemistry analyzer (Beckman Coulter Inc., Brea, CA) using manufacturer's reagents and the method is standardized to the IDMS (isotope dilution mass spectrometry). Besides, the micro total protein assay (M-TP assay, Beckman coulter) was used for the quantitative determination of total urine protein. The M-TP reagent was used to measure protein concerntration by a timed endpoint method. Protein in the sample reacted with the pyrogallol red (PR) molybdate (Mo) complex to form a purple color that has a maximum absorbance at 600 nm. The assays were measured at the Taichung Veterans General Hospital, Taiwan. In addition, the presence of arterial hypertension with blood pressure >140/90 mm Hg, and the presence of ESRF requiring renal replacement therapy (Cockcroft was always below 15 mL/min) were recorded. All patients were treated according to their needs: patients with hypertension received optimal antihypertensive therapy, 113 (87.6%) patients received angiotensin-converting enzyme inhibitors (ACEIs) or angiotensin II receptor blockers (ARBs) for heavy proteinurea, and 30 (23.3%) patients received prednisolone. Some patients received more than 1 treatment during the disease. The response and outcomes were estimated by measuring the serum creatinine (Cr) and the total urine protein as mentioned above. The responses to therapy were defined as (1) no response; (2) partial remission, defined as a proteinuria reduction of >50% or a final proteinuria level between 0.2 and 2.0 g/day. (3) complete remission, defined as proteinuria < 0.2 g/day. Progression of renal disease was defined as a doubling of baseline serum Cr values or ESRF. ESRF was defined as an irreversible decline in kidney function requiring renal replacement therapy. The inclusion criteria are as follows: (1) individual must satisfy the diagnostic criteria of IMN at the time of examination; (2) individual is willing to participate and is capable of giving informed consent; and (3) individual is a self-reported non-aboriginal Taiwanese, and none of the parents and grandparents has aboriginal background. The exclusion criteria are as follows: (1) individual is unable to understand or give informed consent or (2) individual is pregnant or had childbirth within 1 year.

#### Controls

The control group was matched for gender (64 male individuals [60.4%] and 42 female individuals [39.6%]) in accordance with the predominance of IMN in men. The average age was different in controls (27.3 ± 6.6 years) compared with IMN patients (57.0 ± 16.9 years) (*χ*^2 ^= 194.140, *p *= 2.770 × 10^-15^). The average BMI of controls was 21.8 (± 3.1).

### Single nucleotide polymorphism selection

The genotype information of *PLA2R1 *single nucleotide polymorphism (SNP) was downloaded in December 2008 from the HapMap CHB + JPT population. HapMap genotypes were analyzed with Haploview, and Tag SNPs were selected using the Tagger function by applying the following additional criteria: (1) a threshold minor allele frequency in the HapMap CHB + JPT population of 0.05 for tag SNPs" and (2) probe/primers that pass the qualification as recommended by the manufacturer (Applied Biosystems), to ensure a high genotyping success rate. One polymorphism that met the criteria, SNP rs6757188 (*C*/*T*) in intron 12, and a polymorphism that results in amino acid change (His to Asp), SNP rs35771982 (*C*/*G*) in exon 5 of the gene, were selected.

### Genomic DNA extraction and genotyping

All samples from individuals were collected, by venipuncture, for subsequent genomic DNA isolation. Genomic DNA was extracted from peripheral blood leukocytes using a genomic DNA kit (Qiagen) according to the manufacturer's instructions. Genotyping was achieved using an assay-on-demand allelic discrimination assay and detection system according to the manufacturer's instructions (Applied Biosystems). The reaction mixture for the real-time polymerase chain reaction (PCR) contained 10 ng of genomic DNA, 10 μL TaqMan master mix, and 0.125 μL of 40× assay mix. PCR was performed in 96-well plates on a thermal cycler (ABI 7700; Applied Biosystems). Reaction conditions were 50°C for 2 min and 95°C for 10 min, followed by 40 cycles at 95°C for 15 s and 60°C for 1 min. To confirm the quality and accuracy of genotyping data from TaqMan assay, PCR-restriction fragment length polymorphism (RFLP) was performed. Amplification reaction was performed at the total volume of 20 μL using a thermal cycler (ABI 9700; Applied Biosystems). Reaction mixtures contained 100 ng genomic DNA, 10 pmol of each primer, 0.25 mmol of dNTPs, 0.5 U ExTaq polymerase (Takara), and PCR buffer.

**Figure 1:**
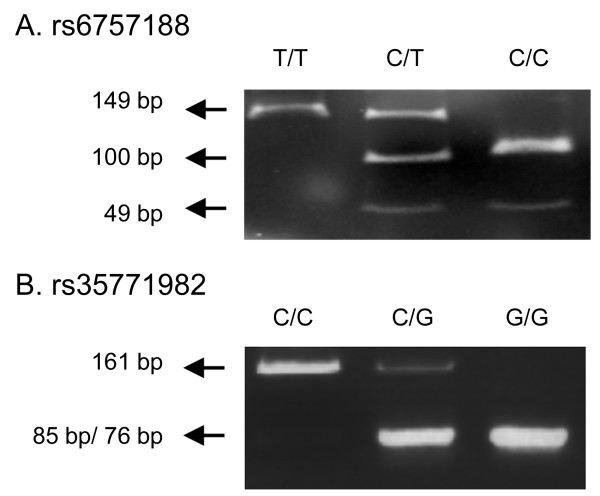
**Identification of *phospholipase A2 receptor 1 *(*PLA2R1*) rs6757188 and rs35771982 genotypes by using polymerase chain reaction-restriction fragment length polymorphism (PCR-RFLP)**. (A) *HpyCH*4III restriction enzyme digestion of the intron region at rs6757188. Lanes 1: DNA from an individual homozygous for the polymorphic allele of *T/T*. Lane 2: DNA from an individual homozygous for the polymorphic allele of *C/T*. Lane 3: DNA from an individual heterozygous for the polymorphic allele of *C/C*. (B) *Bbs*I restriction enzyme digestion of the exon region at rs35771982. Lane 1: DNA from an individual homozygous for the polymorphic allele of *C/C*. Lane 2: DNA from an individual homozygous for the polymorphic allele of *C/G*. Lane 3: DNA from an individual heterozygous for the polymorphic allele of *G/G*.

***rs6757188***. The following primers were constructed: forward primer 5'-*AAAGGGCCCCGGAATAAAGGAA*-3' and reverse primer 5'-*TTTCACCCCTG-CTATTTGGACTG*-3'. The PCR product (149 bp) was digested with the *HpyCH*4III restriction endonuclease enzyme (New England Biolabs, NEB) at 37°C for 4 h followed by 3.5% agarose gel electrophoresis analysis. *HpyCH*4III digestion of the PCR product yielded 49 bp and 100 bp fragments for the *C *allele, whereas a single 149 bp fragment was observed for the *T *allele (Fig. 1A).

***rs35771982*. **The following primers were constructed: forward primer 5'-*GAAGCTCCATAATTTTCATTTCAGAGC*-3' and reverse primer 5'-*GGCAAAGAAAACACTGCGGGTA*-3'. The PCR product (161 bp) was digested with the *Bbs*I restriction endonuclease enzyme (NEB) at 37°C for 4 h followed by 3.5% agarose gel electrophoresis analysis. *Bbs*I digestion of the PCR product yielded 85 bp and 75 bp fragments for the *G *allele, whereas a single 161 bp fragment was observed for the *C *allele (Fig. 1B).

### Statistical analysis

The allelic frequency and genotype frequency distributions of the polymorphisms in individuals with or without IMN, or in IMN patients with several clinical features, were analyzed by *χ*^2 ^test, ANOVA test, or Mantel-Haenszel test. The odds ratio (OR) was calculated from genotype and allelic frequencies with a 95% confidence interval (95% CI). Haplotypes were inferred from unphased genotype data using the Phase 2.1 program based on the Bayesian algorithm [14]. Pairwise linkage disequilibrium (LD) coefficients (r^2^) between SNPs were calculated to evaluate the structure of LD in IMN patients and controls. The Kaplan-Meier method was used to estimate cumulative survival, the results of which were used to investigate the significant factors influencing ESRF/death. SPSS Version 18.0 software was used to analyze the data.

## Results

### *PLA2R1 *gene polymorphisms in IMN patients and in controls

Two SNPs within *PLA2R1 *were genotyped in 129 patients with IMN and in 106 healthy controls. Analysis of IMN patients in comparison with controls revealed a significant difference in allelic distributions for the exon 5 SNP rs35771982 (*p = *0.005; OR_allele *G*/allele *C*_, 1.900; 95% CI, 1.209-2.987). The significance remained after applying the Bonferroni correction. The distribution of SNP rs6757188 did not exhibit a significant association with the disease. The effect of each allele was evident for the genotype distributions (Table 1). These results demonstrate that the *G *allele and the *G/G *genotype of SNP rs35771982 may increase the incidence of IMN.

**Table 1:** Genotype and allele frequencies of *phospholipase A2 receptor 1 *(*PLA2R1*) single nucleotide polymorphisms (SNPs) in patients with idiopathic membranous nephropathy (IMN) vs healthy controls

SNPs	Controls N (%)	IMN N (%)	***p*-Value**^**a**^	**Odds ratio (95% CI)**^**b**^
**Alleles**				
rs6757188				
*C *allele	76 (35.8)	83 (32.3)	0.402	1
*T *allele	136 (64.2)	175 (67.8)		1.178 (0.803-1.729)
Total	212 (100)	258 (100)		
rs35771982				
*C *allele	56 (26.4)	41 (15.9)	0.005	1
*G *allele	156 (73.6)	217 (84.1)		1.900 (1.209-2.987)
Total	212 (100)	258 (100)		

**Genotypes**				
rs6757188				
*C*/*C*	9 (8.5)	15 (11.6)	0.113	1
*C*/*T*	58 (54.7)	53 (41.1)		0.548 (0.221-1.357)
*T*/*T*	39 (36.8)	61 (47.3)		0.938 (0.374-2.352)
Total	106 (100)	129 (100)		
rs35771982				
*C*/*C*	8 (7.5)	2 (1.6)	0.015	1
*C*/*G*	40 (37.7)	37 (28.7)		3.700 (0.738-18.560)
*G*/*G*	58 (54.7)	90 (69.7)		6.207 (1.273-30.262)
Total	106 (100)	129 (100)		

Abbreviation: CI, confidence interval.

^a ^Genotype frequencies were determined by *χ*^2 ^test using 2 × 3 contingency tables between patients with IMN and healthy controls. Allele frequencies and restricted genotypes were determined by *χ*^2 ^test using 2 × 2 contingency tables between patients with IMN and healthy controls.

^b ^Odds ratios and 95% CI per genotype and allele were estimated by applying unconditional logistic regression between patients with IMN and healthy controls.

### Haplotype analysis of *PLA2R1*

All the haplotypes presented in our study are shown in Table 2. Comparisons of the haplotype frequencies between patients with IMN and controls revealed significant differences (*p *= 0.004). Patients with IMN showed an increase in frequency of haplotype H1 (OR = 1.647, 95% CI = 1.140-2.379). In addition, patients with IMN also showed a decrease in frequencies of haplotype H3 (OR = 0.581, 95% CI = 0.368-0.919). These observations suggest that H1 might increase the risk of development of IMN, and H3 might decrease this risk.

**Table 2:** Haplotypes according to the presence of *phospholipase A2 receptor 1 *(*PLA2R1*) single nucleotide polymorphisms (SNPs) in patients with idiopathic membranous nephropathy (IMN) vs healthy controls

Polymorphisms	Controls N (%)	IMN N (%)	*p*-Value	**Odds ratio (95% CI)**^**e**^
**Haplotype**^**a**^				
H1-*TG*	84 (39.6)	134 (51.9)	0.004^b^	1.647 (1.140-2.379)
H2-*CG*	72 (34.0)	83 (32.2)		0.922 (0.627-1.357)
H3-*TC*	52 (24.5)	41 (15.9)		0.581 (0.368-0.919)
H4-*CC*	4 (1.9)	0 (0.0)		0.005 (0.000-)

**Diplotypes**^**a**^				
H1/H1 + H1/nonH1	**67 (63.2)**	**99 (76.7)**	0.023^c^	**1.921 (1.088-3.390)**
H1/H1	17 (16.0)	35 (27.1)	0.031^d^	2.676 (1.264-5.665)
H1/nonH1	54 (47.2)	64 (49.6)		1.664 (0.911-3.041)
nonH1/nonH1	39 (36.8)	30 (23.3)		1
				
H2/H2 + H2/nonH2	64 (60.4)	68 (52.7)	0.239^c^	**1.921 (1.088-3.390)**
H2/H2	8 (7.5)	15 (11.6)	0.174^d^	2.676 (1.264-5.665)
H2/nonH2	56 (52.8)	53 (41.1)		1.664 (0.911-3.041)
nonH2/nonH2	42 (39.6)	61 (47.3)		1
				
H3/H3 + H3/nonH3	47 (44.3)	39 (30.2)	0.025^c^	0.544 (0.318-0.930)
H3/H3	5 (4.7)	2 (1.6)	0.053^d^	0.262 (0.049-1.396)
H3/nonH3	42 (39.6)	37 (28.7)		0.578 (0.333-1.002)
nonH3/nonH3	59 (55.7)	90 (69.8)		1
				
H4/H4 + H4/nonH4	4 (3.8)	0 (0.0)	0.026^c^	0.000 (0.000-)
H4/H4	0 (0.0)	0 (0.0)	0.026^d^	--
H4/nonH4	4 (3.8)	0 (0.0)		0.000 (0.000-)
nonH4/nonH4	102 (96.2)	129 (100.0)		1

Abbreviations: CI, confidence interval; *H*, haplotype.

^a ^Order of SNPs comprising the *PLA2R1 *haplotypes: rs6757188, and rs35771982. The haplotypes were identified by the Baysian statistical method available in the program Phase 2.1.

^b ^The significance of haplotype frequency for IMN development was determined by chi-square test using 4 × 2 contingency tables.

^c ^Individual haplotype frequencies for IMN development were determined by chi-square test using 2 × 2 contingency tables.

^d ^The significance of diplotype frequency for IMN development were determined by chi-square test using 3 × 2 contingency tables.

^e ^Odds ratios and 95% CI per haplotype or diplotype were estimated by applying unconditional logistic regression between patients with IMN and healthy controls.

The 235 individuals for whom *PLA2R1 *haplotypes were evaluated were divided into 3 groups according to their diplotypes (Table 2). The significance of haplotype H1 and H3 in diplotype analysis remained, although did not meet the Bonferroni correction. For haplotype H1, there were 52 homozygous haplotype H1 carriers (H1/H1), 118 heterozygous haplotype H1 carriers (H1/nonH1), and 69 individuals lacking haplotype H1 (nonH1/nonH1). Patients with diplotype H1/H1, or with at least one H1 haplotype were associated with a 2.676-and 1.921-fold greater susceptibility for IMN (*p *= 0.031 and 0.023, OR: 2.676 and 1.921, 95% CI 1.264-5.665 and 1.088-3.390, respectively). In addition to H1, patients with at least one haplotype H3 decreased the risk of development of IMN by 54.4% (*p *= 0.025, OR: 0.544, 95% CI 0.318-0.930). These results suggest that H1 may be a risk factor and that H3 may inhibit the development of IMN.

### LD analysis

To clarify the structure of the LD around rs6757188 and rs35771982, r^2 ^values between the 2 SNPs were calculated. LD analysis for the rs6757188-rs35771982 region revealed that the 2 polymorphisms, with or without implication in IMN, were not in LD in both controls and IMN patients (all r^2^ < 0.030).

### Relationship between rs6757188 and rs35771982 polymorphisms and clinical features of IMN

**Figure 2:**
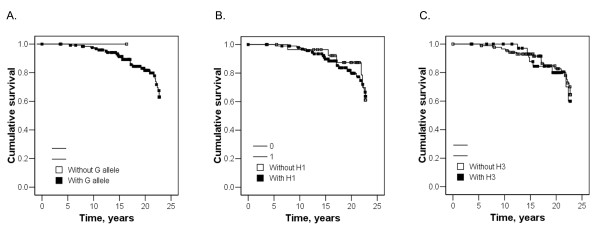
**Risk polymorphism, risk haplotype and protective haplotype of *phospholipase A2 receptor 1 *(*PLA2R1*) and survival without end-stage renal failure (ESRF)/death**. (A) Survival without ESRF/death of individuals with or without the risk *G *allele at rs35771982 of *PLA2R1 *(log rank significance: 0.74). (B) Survival without ESRF/death of individuals with or without the risk haplotype 1 of *PLA2R1 *(log rank significance: 0.77). (C) Survival without ESRF/death of individuals with or without the protective haplotype 3 of *PLA2R1 *(log rank significance: 0.70).

The role of the risk *PLA2R1 *polymorphisms in the progression of disease toward ESRF/death was investigated. Non of the risk allele, the risk haplotype nor the protective haplotype of *PLA2R1 *had an effect on survival without ESRF/death (Fig. 2). Clinical features of IMN patients with genotypes of the 2 *PLA2R1 *polymorphisms are shown (Additional file 1: Table S1). No significant difference was found in gender distribution, age, BMI, systolic blood pressure, diastolic blood pressure, serum albumin level, haematuia or proteinuria. The initial laboratory test reveals no difference in baseline serum Cr level, daily urinary protein excretion (DUP), or creatinine clearance (CCr). After a mean of 6.3 ± 5.1 years follow-up, there was no significant difference in the last measured serum Cr level, last measured urine protein level, or last measured CCr level. In the 104 of 129 cases (80.6%) with clinical-pathological records of biopsies results, the grades of IMN patients were not significantly different among the genotypes of the 2 polymorphisms.

### Relationship between gene polymorphisms and treatment outcomes

Outcomes of IMN patients treated with either supportive or aggressive immunosuppressants were not different among the different genotype groups of rs6757188 and rs35771982 polymorphisms. Mantel-Haenszel test revealed that the genotype *C/T *at rs6757188 and the genotype with *C/G *at rs35771982 were associated with a low rate of remission during disease progression after therapy (Additional file 2: Table S2).

### *The risk PLA2R1 *polymorphisms did not associated with ESRF/death

The role of the risk *PLA2R1 *polymorphisms in the disease progression toward ESRF/death was investigated. None of the risk allele, risk haplotype nor protective haplotype of *PLA2R1 *had an effect on survival without ESRF/death (Fig. 2).

## Discussion

The polymorphisms of several candidate genes, such as *plasminogen activator inhibitor-1 *(*PAI-1*) [15], *tumor necrosis factor A *(*TNFA*) [16], *metallomembrane endopeptidase *(*MME*) [17], *major histocompatibility complex (MHC) class II *[18], and *complement factor B subtype FA *(FB*FA) [19], have been reported to contribute to IMN. Although *PLA2R1 *has been proposed to represent the target autoantigen for IMN [13], the occurrence of *PLA2R1*polymorphisms in IMN patients has not been investigated. In this study, we investigated 2 SNPs (rs6757188 and rs35771982) in *PLA2R1 *and their association with IMN. Our results suggest that the G allele at SNP rs35771982 in exon 5 may increase this risk of this disease. Although the disease-associated *PLA2R1 *haplotype may not be limited to the SNPs we have analyzed, our data demonstrate that the haplotype H1-*TG *may represent a susceptibility haplotype for IMN, while H3-*TC *may be a protective haplotype. To our knowledge, this is the first study to demonstrate that *PLA2R1 *polymorphisms may be associated with the development of IMN. These findings suggest that *PLA2R1 *may play a pathogenic role in inducing or maintaining glomerular barrier dysfunction in humans.

The role of PLA2R1 in the pathogenesis of IMN is supported by evidence of high urinary levels of this membranous protein and the significant presence of PLA2R1 antibodies in patients with IMN [13]. To date, however, its role in the development of IMN has not been elucidated. The *PLA2R1 *gene is located on chromosome 2q23-q24 [12]. Polymorphisms in *PLA2R1 *may have a direct effect on the gene function, but this has rarely been discussed. PLA2R1 is naturally expressed on the cell membrane of podocytes and acts as a receptor for sPLA2. This receptor participates in both positive and negative regulation of sPLA2, which is involved in the induction of cell proliferation, cell adhesion, production of lipid mediators, and release of arachidonic acid [11]. Our findings demonstrate that the *G *allele of SNP rs35771982 in exon 5, which results in a residue change (H300D) in the second of the 8 CTLDs, appears to be more selectively expressed in IMN patients than in controls. CTLDs are involved in several functions, such as extracellular matrix organization, endocytosis, complement activation, pathogen recognition, and cell-cell interactions [11,20,21]. Alterations in the structures of these domains may affect these important functions. Overproduction of serum PLA2R1 antibodies that has been observed in individuals may be linked to the *A*/*G *allele of SNP rs35771982. Although the intron 12 SNP rs6757188 of *PLA2R1 *was not statistically linked to the development of IMN, the *T *allele of rs6757188 has a protective effect on the development of global sclerosis and tubulointerstitial fibrosis (data not shown). Confirmation of these results in larger samples is warranted.

One aim of genetic studies is to provide information about the prognosis of a given disease. IMN exhibits large interindividual variations in the progression toward ESRF/death. Aggressive therapies with steroids and immunosuppressive drugs such as Ponticelli's protocol [22] or cyclosporine [23] cause many side effects [9]. Therefore, it is important to identify markers for the early identification of patients who are at risk for the progression of this disease. Indeed, several factors have been identified for predicting poor prognosis [24,25]. However, our investigation of the association between *PLA2R1 *polymorphisms and ESRF/death did not provide genetic information for predicting the risk of ESRF/death.

In addition, a correlation has emerged between plasminogen activator inhibitor-1 *4G *allele and IMN progression. Stratified analysis using the Mantel-Haenszel statistic revealed a high disease progression in *4G4G *genotype patients with no remission of proteinuria [15]. Moreover, the *A/A *genotype for rs401824 and the *G/G *genotype for rs437168 of the *NPHS1 *gene show correlation with no remission of proteinuria [26]. Similarly, we observed disease progression in the *C/T *genotype for rs6757188, and the *C/G *genotype for rs35771982 showed correlation with a low rate of remission of proteinuria. Most of the patients in this study were treated with ACEIs and ARBs. Despite the similar mode of treatment given to our patients, more disease progression was found in patients with *C/T *genotype of rs6757188 as well as *C/G *genotype of rs35771982 than in other subgroups, suggesting that more specific drugs targeting biosynthesis or activity of PLA2R1 may supplement regular immunosuppressive regimens, particularly in patients with *C/T *genotype of rs6757188 and *C/G *genotype of rs35771982.

## Conclusions

This study provides evidence that genetic factors are responsible for the development and progression of IMN. Our results suggest that the presence of *G *allele at the *PLA2R1 *SNP rs35771982 and the H1-*TG *haplotype may initiate, while the H3-*TC *may decrease the risk of IMN. In addition, the presence of *C/T *genotype of rs6757188 and *C/G *genotype of rs35771982 leads to a low rate of remission during IMN progression after therapy. This study provides evidence that SNPs in the *PLA2R1 *gene are associated with the risk of development and progression of IMN. These results might aid diagnosis during the early stage of disease and may be valuable for therapeutic studies in the Taiwanese population.

## List of abbreviations

(*PLA2R1*): *Phospholipase A2 receptor 1*; (IMN): idiopathic membranous nephropathy; (MN): membranous nephropathy; (SNPs): single nucleotide polymorphisms; (ESRF/death): end-stage renal failure and death.

## Competing interests

The authors declare that they have no competing interests.

## Authors' contributions

LYH designed the study, managed the literature searches, performed the SNP experiments, undertook the statistical analysis, and wrote the draft of the manuscript. CCH, CSY and LYJ recruited and maintained the clinical information of participants. LWL undertook the statistical analysis.TCH, WL and TFJ directed the study and reviewed the results. All authors contributed to and have approved the final manuscript.

## Supplementary Material

Additional file 1 **Table S1: Comparison of clinical and biochemical manifestations in idiopathic membranous nephropathy (IMN) patients with different *phospholipase A2 receptor 1 *(*PLA2R1*) genotypes distributions of rs6757188 and rs35771982**. The gender distribution, age, BMI, systolic blood pressure, diastolic blood pressure, serum albumin level, haematuia or proteinuria as well as the serum creatinine level, daily urinary protein excretion, or creatinine clearance before and after a mean of 6.3 ± 5.1 years follow-up were no significant difference among the genotypes of the 2 polymorphisms. Click here for file

Additional file 2 **Table S2: Stratified analysis of during disease progression according to *phospholipase A2 receptor 1 *(*PLA2R1*) gene polymorphisms**. The genotype *C/T *at rs6757188 and the genotype with *C/G *at rs35771982 were associated with a low rate of remission during disease progression after therapy. Click here for file
