# Supplementary material for: Association of phospholipase A2 receptor 1 polymorphisms with idiopathic membranous nephropathy in Chinese patients in Taiwan
Source: J Biomed Sci. 2010 Oct 11;17(1):81. doi: 10.1186/1423-0127-17-81 (PMC2959017; doi:10.1186/1423-0127-17-81)
Supplement: Additional file 1 — Table S1: Comparison of clinical and biochemical manifestations in idiopathic membranous nephropathy (IMN) patients with different phospholipase A2 receptor 1 (PLA2R1) genotypes distributions of rs6757188 and rs35771982. The gender distribution, age, BMI, systolic blood pressure, diastolic blood pressure, serum albumin level, haematuia or proteinuria as well as the serum creatinine level, daily urinary protein excretion, or creatinine clearance before and after a mean of 6.3 ± 5.1 years follow-up were no significant difference among the genotypes of the 2 polymorphisms. [file 1423-0127-17-81-S1.DOC]

**Additional file 1.**

**Table S1. Comparison of clinical and biochemical manifestations in idiopathic membranous nephropathy (IMN) patients with different *phospholipase A2 receptor 1* (*PLA2R1*) genotypes distributions of rs6757188 and rs35771982.**

| **Clinical parameters** | **Patients with IMN** | | | | | | | |
| --- | --- | --- | --- | --- | --- | --- | --- | --- |
|  | **rs6757188** | | | | **rs35771982** | | | |
|  | ***CC*** | ***CT*** | ***TT*** | ***p*-Valu*e*** | ***CC*** | ***CG*** | ***GG*** | ***p*-Value** |
| Gender (M/F) | 11/4 | 26/27 | 36/25 | NS ^a^ | 2/0 | 15/22 | 56/34 | 0.037 ^a^ |
| Age (years) | 65.4 ± 15.2 | 54.1 ± 17.3 | 57.7 ± 16.4 | NS ^b^ | 46.3 ± 19.8 | 51.6 ± 19.7 | 59.6 ± 16.4 | NS ^b^ |
| BMI (kg/M 2 ) | 24.0 ± 3.8 | 25.2 ± 3.7 | 24.7 ± 3.4 | NS ^b^ | 23.7 ± 1.6 | 24.9 ± 3.4 | 24.8 ± 3.7 | NS ^b^ |
| SBP (mmHg) | 141.3 ± 30.7 | 132.9 ± 18.1 | 136.3 ± 19.0 | NS ^b^ | 130.0 ± 14.1 | 133.5 ± 20.6 | 136.5 ± 20.4 | NS ^b^ |
| DBP (mmHg) | 80.8 ± 15.3 | 83.0 ± 10.2 | 82.6 ± 13.0 | NS ^b^ | 81.0 ± 12.7 | 82.4 ± 13.4 | 82.6 ± 11.7 | NS ^b^ |
| Baseline serum Cr (mg/dL) | 2.1 ± 2.2 | 1.5 ± 1.5 | 1.3 ± 0.8 | NS ^b^ | 1.5 ± 0.1 | 1.3 ± 1.5 | 1.5 ± 1.3 | NS ^b^ |
| Baseline DUP (g/day) | 6.8 ± 5.9 | 8.3 ± 9.8 | 7.6 ± 10.6 | NS ^b^ | 11.0 ± 2.8 | 5.1 ± 4.0 | 8.8 ± 11.2 | NS ^b^ |
| Baseline CCr (mL/min) | 83.6 ± 52.8 | 79.4 ± 43.0 | 82.4 ± 35.7 | NS ^b^ | 62.0 ± 31.1 | 93.3 ± 40.1 | 76.7 ± 40.5 | NS ^b^ |
| Duration follow-up (years) | 7.2 ± 6.2 | 5.8 ± 4.9 | 6.5 ± 5.0 | NS ^b^ | 12.7 ± 9.0 | 5.5 ± 4.3 | 6.5 ± 5.2 | NS ^b^ |
| Last serum Cr (mg/dL) | 2.2 ± 1.6 | 2.6 ± 2.8 | 3.2 ± 4.6 | NS ^b^ | 2.4 ± 1.3 | 2.4 ± 2.5 | 3.0 ± 4.0 | NS ^b^ |
| Last DUP (g/day) | 1.8 ± 3.6 | 4.1 ± 5.5 | 2.5 ± 3.1 | NS ^b^ | 1.7 ± 2.1 | 2.8 ± 3.4 | 3.3 ± 4.8 | NS ^b^ |
| Last CCr (mL/min) | 47.8 ± 32.9 | 61.6 ± 43.3 | 55.4 ± 41.0 | NS ^b^ | 63.5 ± 47.6 | 64.3 ± 42.4 | 54.3 ± 40.6 | NS ^b^ |
| Cholesterol (mg/dL) | 331.4 ± 122.7 | 307.1 ± 103.4 | 345.8 ± 143.4 | NS ^b^ | 508.5 ± 290.6 | 324.7 ± 119.6 | 325.6 ± 124.4 | NS ^b^ |
| Triglyceride (mg/dL) | 193.0 ± 165.4 | 218.0 ± 144.8 | 236.1 ± 162.8 | NS ^b^ | 222.0 ± 140.0 | 210.0 ± 149.5 | 229.2 ± 159.2 | NS ^b^ |
| Albumin (g/dL) | 2.2 ± 0.4 | 2.5 ± 0.6 | 2.6 ± 0.7 | NS ^b^ | 2.2 ± 0.9 | 2.6 ± 0.6 | 2.5 ± 0.6 | NS ^b^ |
| Clinical presentation |  |  |  |  |  |  |  |  |
| With proteinuria [N, (%)] | 14 (93.3) | 48 (90.6) | 59 (96.7) | NS ^a^ | 2 (100.0) | 37 (100.0 | 82 (91.1) | NS ^a^ |
| With haematuria [N, (%)] | 8 (53.3) | 35 (66.0) | 38 (62.3) | NS ^a^ | 0 (0.0) | 26 (70.3) | 55 (61.1) | 0.018 ^a^ |
| Grade [N, (%)] |  |  |  |  |  |  |  |  |
| 1 | 2 (20.0) | 12 (26.1) | 12 (25.0) | NS ^a^ | 0 (0.0) | 10 (34.5) | 16 (21.9) | NS ^a^ |
| 2 | 4 (40.0) | 23 (50.0) | 28 (58.3) |  | 2 (100.0) | 10 (34.5) | 43 (58.9) |  |
| 3 | 3 (30.0) | 7 (15.2) | 6 (12.5) |  | 0 (0.0) | 7 (24.1) | 8 (11.0) |  |
| 4 | 1 (10.0) | 3 (6.5) | 1 (2.1) |  | 0 (0.0) | 2 (6.9) | 4 (5.5) |  |
| 5 | 0 (0.0) | 1 (2.2) | 1 (2.1) |  | 0 (0.0) | 0 (0.0) | 2 (2.7) |  |
| ^a^ *χ*^2^ test.  ^b^ ANOVA test.  The *p-*values less than 0.05 were considered significant. Significant values are indicated in bold letters. | | | | | | | | |
