# Supplementary material for: Association of phospholipase A2 receptor 1 polymorphisms with idiopathic membranous nephropathy in Chinese patients in Taiwan
Source: J Biomed Sci. 2010 Oct 11;17(1):81. doi: 10.1186/1423-0127-17-81 (PMC2959017; doi:10.1186/1423-0127-17-81)
Supplement: Additional file 2 — Table S2: Stratified analysis of during disease progression according to phospholipase A2 receptor 1 (PLA2R1) gene polymorphisms. The genotype C/T at rs6757188 and the genotype with C/G at rs35771982 were associated with a low rate of remission during disease progression after therapy. [file 1423-0127-17-81-S2.DOC]

**Additional file 2.**

**Table S2. Stratified analysis of during disease progression according to *phospholipase A2 receptor 1* (*PLA2R1*)** gene polymorphisms.

| **Polymorphisms** | **Remission of proteinuria** | **Progression** | | ***p*-Value ^a^** |
| --- | --- | --- | --- | --- |
|  |  | **Non-progression**  **N (%)** | **Progression**  **N (%)** |  |
| rs6757188 |  |  |  |  |
| *CC* | No-remission | 1 (100) | 2 (66.7) | 0.505 |
|  | Remission | 0 (0.0) | 1 (33.3) |  |
| *CT* | No-remission | 5 (55.6) | 7 (100.0) | **0.042** |
|  | Remission | 4 (44.4) | 0 (0.0) |  |
| *TT* | No-remission | 2 (25.0) | 3 (75.0) | 0.098 |
|  | Remission | 6 (75.0) | 1 (25.0) |  |
| rs35771982 |  |  |  |  |
| *CC* | No-remission | 0 (0.0) | 0 (0.0) | -- |
|  | Remission | 0 (0.0) | 0 (0.0) |  |
| *CG* | No-remission | 1 (20.0) | 4 (100.0) | **0.016** |
|  | Remission | 4 (80.0) | 0 (0.0) |  |
| *GG* | No-remission | 7 (53.8) | 8 (80.0) | 0.192 |
|  | Remission | 6 (46.2) | 2 (20.0) |  |
| ^a^ Mantel-Haenszel test.  The *p-*values less than 0.05 were considered significant. Significant values are indicated in bold letters. | | | | |
